# Diagnostic Performance of Computed Tomography-Based Machine Learning Models in the Classification of Adnexal Masses - A Systematic Review

**DOI:** 10.12688/f1000research.178239.1

**Published:** 2026-04-02

**Authors:** Suvarna Kotian, Priyanka -, Varsha R, Rajagopal Kadavigere, Saikiran Pendem, Kaushik Nayak

**Affiliations:** 1 Department of Medical Imaging Technology, Manipal College of Health Professions, Manipal Academy of Higher Education, Manipal, India; 2 Department of Radiodiagnosis and Imaging, Kasturba Medical College, Manipal Academy of Higher Education, Manipal, India

**Keywords:** Computed tomography, adnexal masses, machine learning model

## Abstract

**Introduction:**

Accurate characterization of adnexal masses is a key issue and a crucial step toward improving the outcome of managing a patient with a gynecologic oncology issue. Though ultrasound is a dominant tool for this process, it is subjected to operator variability and is less reliable from a diagnostic perspective. Advances in computed tomography-based radiomics and ML hold great promise as objective diagnostic solutions.

**Methods:**

This systematic review was performed according to the guidelines suggested by PRISMA. The literature research using PubMed, Embase, Scopus, and Web of Science databases included studies that examined CT-based radiomics and ML model performances for classification of adnexal masses and reported diagnostic performance metrics, including AUC, sensitivity, and specificity. Quality assessment of included studies was performed using the QUADAS 2 tool.

**Results:**

Eleven studies were included in the review. The performance of CT-based ML models was found to be moderate to excellent, with an AUC ranging from 0.72 to 0.99. Hybrid radiomics-DL algorithms were found to have a higher performance compared to other algorithms. The studies were found to have low risk of bias.

**Conclusion:**

CT-based radiomics and AI models also hold good prominence as adjunctive tools in differentiating between both benign and malignant adnexal masses and in predicting prognosis.

**PROSPERO registration:** The study has been registered in PROSPERO under the registration number CRD420251266988, on 16 December 2025.

## Introduction

Adnexal masses refer to tumoral formations that originate from the ovaries, fallopian tubes, or the surrounding structures like para-ovarian cysts and polyps, found in females of all ages but especially in the reproductive ages. Also, the masses can originate from functional or non-functional tumors due to physiological changes and inflammatory conditions of benign and malignant neoplasms. ^1^ The potential malignancy underscores the importance of early, precise, and prompt diagnosis to reduce associated morbidity and mortality. ^2^ “Adnexal masses” are commonly found during imaging studies of the pelvis. In some instances, particularly those that are not as common, a mass might present with acute or intermittent pain. In the general population, the prevalence of “adnexal masses” cannot be known since most of the adnexal masses remain asymptomatic and undiagnosed. ^1^

Currently, the difference between benign and malignant “Adnexal masses” is primarily determined by their imaging characteristics. ^3– 5^ Ultrasound (USG) is often used as an imaging modality for the evaluation and characterization of adnexal masses based on non-invasive properties and accessibility. It has limitations in terms of dependency and resolution of inconsistency, affecting its sensitivity for distinguishing between benign and malignant masses. ^6^ CT is frequently utilized in routine clinical practice for the incidental initial detection of conditions due to its spatial resolution, broad accessibility, and shorter acquisition duration. ^7^ The characterization of adnexal masses has traditionally depended upon these imaging modalities: techniques and subjective assessments. Nevertheless, there are limitations in evaluating the heterogeneity of masses. Thus, it is important to use a precise, objective, non-invasive approach for the categorization of adnexal masses using CT imaging as it offers higher sensitivity compared to USG, performs nearly at par with MRI, and provides the additional advantage of rapid acquisition. ^8^

The algorithms used by artificial intelligence (AI) have the ability to scrutinize complex image information and enable the early identification and characterization of lesions using image recognition and the detection of minute details that may not be observable by the human eye. ^9– 13^ However, CT-based AI models have excellent accuracy and specificity in classifying lesion, which helps in cancer imaging and treatment monitoring. ^14– 16^

This review aims to enhance the detection, classification, and characterization of adnexal masses, thereby assisting radiologists in providing more accurate diagnosis. These results may help the gynecologists to choose more appropriate and personalized therapeutic approaches that could improve clinical outcomes and reduce disease aggressiveness in patients with adnexal masses.

## Methods

### Search strategies

This systematic review was performed in accordance with the Preferred Reporting Items for Systematic Reviews and Meta-Analyses (PRISMA) guidelines. ^17^ The protocol was registered in the International Prospective Register of Systematic Reviews (PROSPERO), and the checklist is available in supplementary file 1. Ethical approval was not required as this study analysed previously published articles for which approvals had already been obtained.

### Databases and search strategy

This literature review search was conducted using four databases such as PubMed, Embase, Scopus, and Web of Science which included the following keywords: adnexal masses, ovarian lesions, machine learning models, radiomics and computed tomography. The detailed search strategy and Boolean operator combinations are given in supplementary file 2.

### Study selection

This review includes both prospective and retrospective studies of adnexal masses, mainly assessing the diagnostic, staging and prognosis of lesions through computed tomographic imaging and radiomics models. Original research articles that are ethically approved from the respective institutions and from peer-reviewed journals containing enough amount of automatic segmentation using radiomics models were included. Reviews, editorials, conference abstracts, nonhuman studies, case reports, small case series with fewer than 10 participants, and studies concerning predictive modelling were excluded.

### Data extraction

Two reviewers independently performed the literature screening. The duplicate articles were removed using Rayyan. ^18^ Full texts of potentially relevant articles were retrieved and reviewed in detail. Discrepancies were resolved through discussion with a third reviewer. We also developed an extraction template to standardize extraction by including information on the studies, their participants, and diagnostic performance measures like AUC, sensitivity, and specificity. Meta-analysis was not performed in this review due to the heterogeneity between the models used in the included studies.

### Risk of bias assessment

The quality and risk of bias of the studies were independently evaluated using the quality assessment of diagnostic accuracy studies 2 (QUADAS-2) ^19^ tool. This established framework looks at potential bias and applicability issues in four main areas: patient selection, index test, reference standard, and flow and timing. Each area was rated as having low, high, or unclear risk of bias. This process ensured a clear and organized evaluation of the studies’ validity and clinical importance.

## Results

### Selections approaches

**Figure 1.**
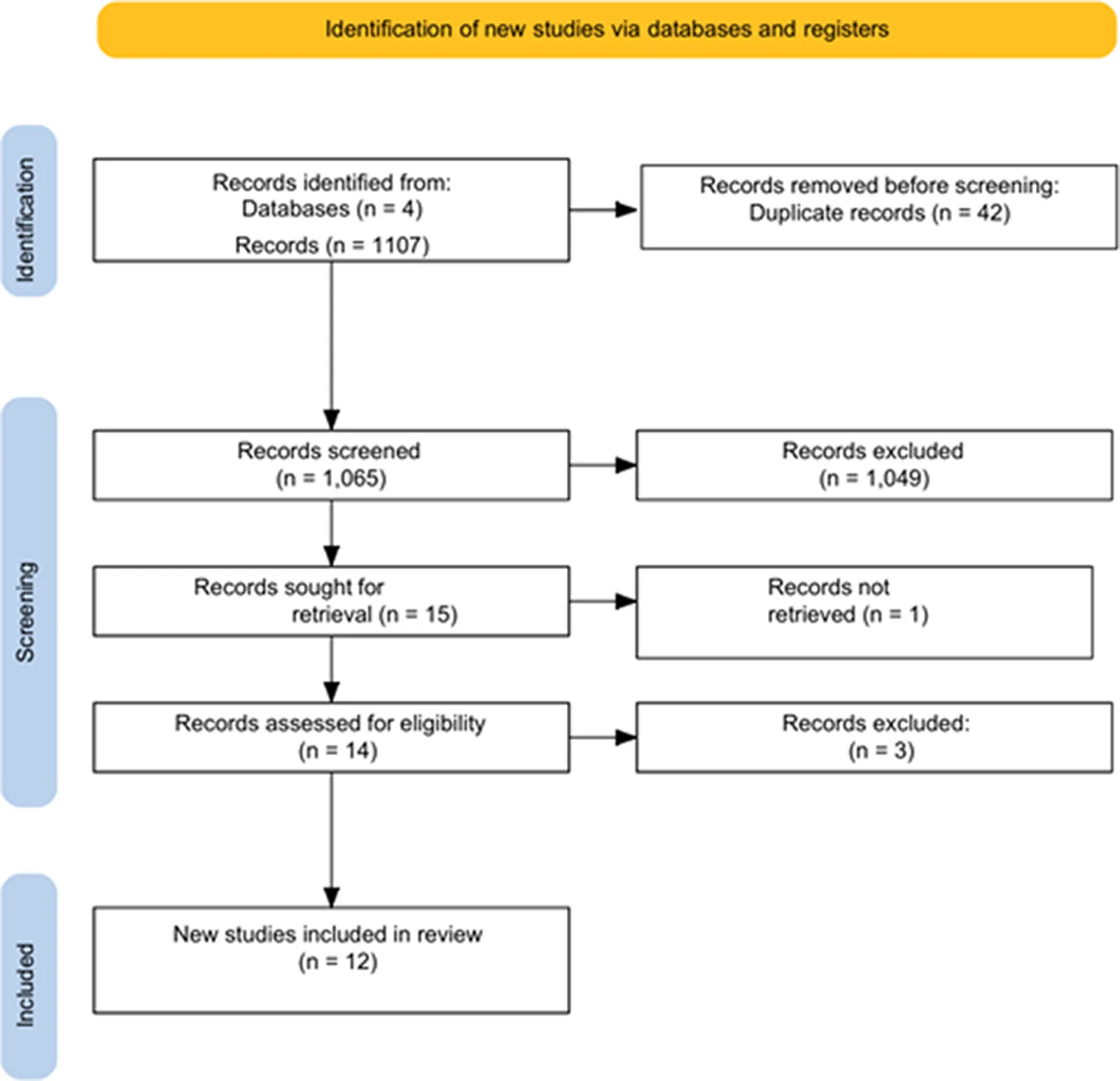
PRISMA flow chart for the articles included in the review.

After duplicate and abstract removal, a total of 1107 original studies were retrieved, and 12 were found to be eligible for full-text screening. Of these, articles are part of the review that fulfilled the inclusion criteria. This process of selection is elaborated in Figure 1.

### Study characteristics

This systematic review combines the results of 11 retrospective studies on CT radiomics, and machine learning published between 2021 and 2025, with a total sample of 4439 patients. The sample size of each study varied from 149 to 1329 patients, and one study included 185 tumors. The studies were mostly carried out in chine (n = 9), with two multicenter studies including patients from the UK, Germany, USA. ^20– 23^ Most of the studies were single -center studies (n = 6), with five studies including two to three centers, which improved external validity.

Clinical tasks included differentiation of benign and malignant ovarian tumors (n = 4), differentiation of serous borderline and malignant tumors (n = 2), prediction of FIGO stage (n = 1), detection of peritoneal metastases (n = 1), and prediction of overall survival (n = 2). All studies were performed using contrast -enhanced CT scans, mainly in the portal venous phase, with 3D VOI segmentation in most cases.

Machine learning algorithms used were logistic regression, support vector machine, random forest, K-nearest neighbors, XGBoost, LightGBM, and deep learning models like CNN and U-Net networks. Validation methods used were train-test split validation, internal validation, leave-one-out cross-validation, and external multi-cohort validation. The diagnostic performance reported was excellent, with AUC ranging from 0.79 to 0.96, accuracy of up to 87%, specificity of up to 89%, and prognostic C-index of up to 0.73, thereby confirming the stability of CT-based radiomics models for the characterization of ovarian cancer. The detailed study characteristics of articles included in the review are provided in Table 1.

**Table 1.** Characteristics of the reviewed studies.

Author (s)	Country	No. centers	Sample size	Group (lesion type)	AI models	Outcome
Yu et al., 2021 ^21^	China	1	182 patients	Serous borderline vs serous malignant tumors	Radiomics + SVM classifier	Best AUC 0.86 (Venous phase)
Li et al., 2022 ^22^	China	3	1329 patients	Benign vs malignant ovarian tumors	Radiomics + ML (KNN, SVM, RF, LR, MLP, XGBoost); best: MLP	Mixed model AUC 0.96; Accuracy 0.87
Jan et al., 2023 ^28^	Taiwan	1	149 patients	Benign vs Malignant ovarian tumors	Radiomics + Deep learning (3D U-Net features) + ML ensemble	Accuracy 82%; Specificity 89%
Li et al., 2023 ^23^	China	3	287 patients	Ovarian cystadenoma vs endometriotic cyst	LASSO + Logistic regression (nomogram)	AUC 0.94 (Validation)
Li et al., 2023 ^25^	China	2	470 patients	Type I vs Type II epithelial ovarian cancer	LR, SVM, RF, KNN, NB, XGBoost	Combined model AUC 0.93
Linton-Reid et al., 2023 ^20^	UK, Germany, USA	3	607 patients	Overall survival (HGSOC)	U-Net + ML radiomics	C-index up to 0.73
Leng et al., 2024 ^27^	China	3	201 patients	FIGO stage (early vs advanced)	LightGBM, LR, SVM, RF, DT	Combined model AUC 0.79 (external)
Chen et al., 2024 ^24^	China	1	258 patients	Benign vs borderline vs early malignant tumors	RF, SVM, LR, KNN, DT	RF AUC 0.81(test)
Yu et al., 2024 ^29^	China	1	182 patients	Early-stage serous borderline vs malignant tumors	Radiomics signature + clinicoradiologiocal nomogram	Nomogram AUC 0.91 (validation)
Su et al., 2025 ^30^	China	2	455 patients	Overall survival prediction	LASSO + Cox ML model	5-yr AUC ≈ 0.87
Liu et al., 2025 ^26^	China	1	296 patients	Peritoneal metastasis (PM)	Radiomics + Deep learning (CNN)	DLRN AUC 0.96

AI- Artificial Intelligence, AUC- Area Under the Receiver Operating Characteristics Curve, CNN- Convolution Neural Network, C-index- Concordance Index, CT- Computed Tomography, DL- Deep Learning, DLRN- Deep Learning Radiomics Network, DT- Decision Tree, FIGO- International Federation of Genecology and Obstetrics, HGSOC- High Grade Serous Ovarian Cancer, KNN- K-Nearest Neighbors, LASSO- Least Absolute Shrinkage and Selection Operator, LightGBM- Light Gradient Boosting Machine, LR- Logistic Regression, ML- Machine Learning, MLP- Multilayer Perception, NB- Naïve Bayers, PM- Peritoneal Metastasis, RF- Random Forest, SVM- Support Vector Machine, U-Net- U-shaped Convolution Neural Network Architecture, XGBoost- Extreme Gradient Boosting.

### Performance accuracy

Among the studies, radiomics and deep learning models showed moderate to excellent diagnostic performance, with AUC values ranging from 0.72 to 0.99 ( Table 2). The highest accuracy was reported by Chen et al., ^24^ achieving an AUC of 0.98 to 0.99. This was followed by Li et al. ^25^ with an AUC of 0.96, and Liu et al. ^26^ with an AUC of 0.951. Liu et al. integrated radiomics with a ResNet-18 deep learning framework. Most studies reported AUC values above 0.85, indicating strong discriminative ability. Sensitivity ranged from 68% to 91.7%. The highest sensitivity was observed in Liu et al. ^26^ at 91.7% and in Li et al. ^23^ at 90%, indicating good detection performance. Specificity varied from 75% to 99%, with Leng et al. ^27^ achieving the highest specificity at 99%. Overall, models that included wavelet-transformed features, higher-order texture metrics, and deep learning architectures consistently achieved better accuracy. This highlights the advantages of improved feature extraction and hybrid radiomics-DL strategies. These findings confirm the high diagnostic potential of radiomics and AI-based models for characterizing lesions, although differences in feature selection, modeling methods, and validation protocols led to varying performance across studies.

**Table 2.** Performance accuracy of the included studies.

Author (s)	Features extracted	Features used	AUC	Sensitivity (%)	Specificity (%)
Yu et al., 2021 ^21^	Shape, first-order, GLCM, GLRLM, GLSZM, NGTDM	9 radiomics features	0.86	80	75
Li et al., 2022 ^22^	Shape, first-order, GLCM, GLRLM, GLSZM, GLDM, NGTDM, LoG, wavelet	Selected raiomics subsets	0.96	81	90
Jan et al., 2023 ^28^	Histogram, GLCM, wavelet, LoG + CNN	Reduced the combined feature set	0.82	68	89
Li et al., 2023 ^23^	Shape, first-order, GLCM, GLRLM, GLSZM, GLDM, NGTDM, wavelet, LoG	17 Radiomics Features	0.925	90	87.7
Li et al., 2023 ^25^	Shape, first-order, texture, wavelet, LoG	Radiomics Signature	0.879	75.6	80.4
Linton-Reid et al., 2023 ^20^	Shape, first-order, texture, wavelet	Optimal reduced radiomics set	0.72	NR	NR
Leng et al., 2024 ^27^	Shape, first-order, GLCM, GLRLM, GLSZM, GLDM, NGTDM, wavelet	7 radiomics features	0.83	84	99
Chen et al., 2024 ^24^	Shape, first-order, texture, wavelet	Reduced radiomics set	0.98–0.99	NR	NR
Yu et al., 2024 ^29^	Shape, first-order, texture	9 radiomics features	0.909	82	84
Su et al., 2025 ^30^	shape, first-order, GLCM, GLRLM, GLSZM, GLDM, NGTDM	Rad-score features	0.816	NR	NR
Liu et al., 2025 ^26^	Radiomics + CNN (ResNet-18)	9 radiomics +10 DL features	0.951	91.7	95.1

AUC- Area Under the Receiver Operating Characteristic Curve, CNN- Convolutional Neural Network, DL- Deep Learning, GLCM- Gray Level Co-occurrence Matrix, GLDM- Gray Level Dependence Matrix, GLRLM- Gray Level Run Length Matrix, GLSZM- Gray Level Size Zone Matrix, LoG- Laplacian of Gaussian, NGTDM- Neighboring Gray Tone Difference Matrix, NR- Not Reported, ResNet-18- Residual Neural Network with 18 layers, Rad-score- Radiomics Score.

### Risk of bias analysis

**Figure 2.**
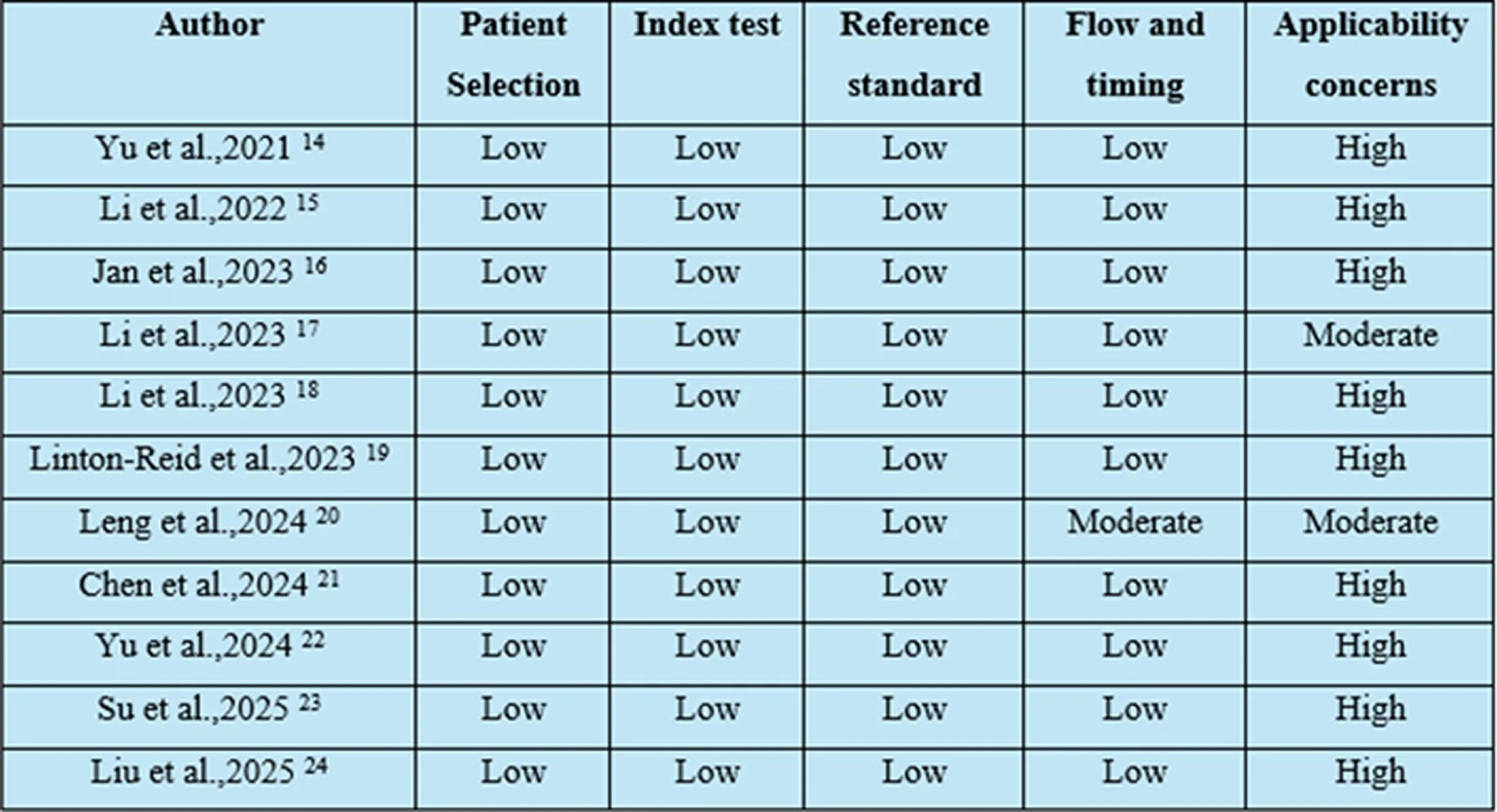
QUADAS-2 analysis.

The quality of the studies included was assessed using the QUADAS-2. Generally, there was a low risk of bias in the domains of patient selection, index test, and reference standard, which is an indication of high methodological quality showed in Figure 2.

All the studies included in the review, namely Yu et al., ^21^ Li et al., ^25^ Jan et al., ^28^ Li et al., ^23^ Linton-Reid et al., ^20^ Leng et al., ^27^ Chen et al., ^24^ Yu et al., ^29^ Su et al., ^30^ and Liu et al., ^26^ had a low risk of bias in the domain of patient selection, which is an indication that the studies had appropriate study populations and that there was no selection bias. The domains of index test and reference standard also had low risks of bias, which is an indication that the studies applied the tests appropriately and that they used accepted diagnostic reference standards. Low risk was found in most studies in the flow and timing domain, reflecting appropriate intervals between the index test and reference standard. However, a moderate risk was found in this domain by Leng et al., ^27^ which could be attributed to differences in follow-up or reporting.

However, concerns regarding applicability were found to be high in most studies, primarily because of the single-center study nature, lack of heterogeneity in the population, and differences in imaging protocols and model validation approaches. Only Li et al. ^23^ and Leng et al. ^27^ reported a moderate level of concerns regarding applicability. Thus, although the internal validity was excellent, external validity is poor, and there is a need for multicenter, externally validated studies.

## Discussion

This systematic review draws attention to the increasing importance of CT radiomics and machine learning models in the evaluation of adnexal masses, especially in differentiating benign from malignant ovarian tumors. In general, most of the models used in the studies had moderate to excellent performance, which indicates that image analysis can provide important information beyond visual inspection.

The vast majority of the included studies used contrast-enhanced CT scans, and there was a strong preference for the portal venous phase, as reported by Yu et al. ^21^ and Li et al. ^31^ The portal venous phase offers more stable lesion enhancement and the ability to visualize tumor heterogeneity, which is essential for radiomics analysis. The studies using this phase reported significantly higher AUC values, as reported in the earlier imaging literature that suggests portal venous CT as the optimal phase for ovarian tumor assessment.

With respect to analytical methods, ensemble or hybrid methods tended to perform better than single algorithm classifiers. Li et al., ^25^ reported that the use of multiple classifiers in ML (random forest, support vector machine, and multi-layer perceptron) resulted in an AUC of 0.96, which was superior to the performance of individual classifiers. Likewise, Li et al. ^23^ showed that the use of nomogram-based methods, which integrated radiomics and logistic regression, was superior in terms of robustness, with a balance between high accuracy and interpretability. By contrast, single-method classifiers like the radiomics-SVM model used by Yu et al. ^21^ tended to perform relatively poorly (AUC 0.86), suggesting a lack of ability to model the complexity of tumors.

The models that integrated deep learning (DL) performed very well in more complex clinical tasks. Liu et al. ^26^ combined the radiomics approach with a ResNet-18 architecture to predict peritoneal metastasis with an AUC of 0.95 and high sensitivity and specificity. Jan et al. ^28^ also combined the 3D U-Net-derived features, showing that the DL approach can extract spatial and hierarchical tumor information that may not be captured by handcrafted radiomics alone. These results are in line with Park et al., ^32^ who found that the combination of CT texture analysis with ML improved the detection of ovarian malignancy compared to radiologist assessment alone.

When contrasted with previous radiomics analysis reviews in the context of ovarian cancer imaging, the results of this review are consistent with the general consensus that tree-based and boosting methods (random forest, XGBoost, LightGBM) generally perform better than simpler distance-based approaches like K-nearest neighbors and naive Bayes. Previous studies that are not included in this review have also highlighted that hybrid clinicoradiomic models generally offer improved diagnostic performance compared to radiomics models alone.

However, some limitations were apparent despite the encouraging results. The majority of the studies were retrospective and single-center, which may pose a risk of selection bias and lack of generalizability. There was heterogeneity in the parameters of CT image acquisition, segmentation approaches (2D vs. 3D), feature selection algorithms, and validation procedures, making it difficult to compare the results and perform meta-analysis. Moreover, some of the models were not externally validated prospectively, which is essential for clinical use.

Future studies should focus on large-scale, prospective, multi-institutional studies with standardized CT acquisition and radiomics pipelines. The use of fully automated segmentation and end-to-end deep learning models may improve clinical applicability. External validation on different populations and scanner platforms is necessary before clinical application. The combination of radiomics analysis with clinical and genomic information may also help in individualized risk assessment and management of adnexal masses.

## Conclusion

CT radiomics and machine learning algorithms have shown great potential as ancillary tools for the assessment of adnexal masses. The algorithms have shown high accuracy and could potentially help radiologists in distinguishing between benign and malignant masses, thus helping in appropriate management. However, before their widespread use, there is a need for further prospective studies. Once validated, these tools could help in improving the accuracy of diagnosis and thus help in personalized management in gynaecologic oncology.

## Ethics and consent

This is a review article. Ethical approval and consent were not required.

## Data Availability

No data is associated with this article. Fig share: Adnexal masses SR. https://doi.org/10.6084/m9.figshare.31332268. ^33^ This project contains the following:
1. Supplementary File 2 (Detailed search strategy) Data are available under the terms of the Creative Commons Attribution 4.0 International license (CC-BY 4.0). Fig share: PRISMA 2020 for Diagnostic Performance of Computed Tomography-Based Machine Learning Models in the Classification of Adnexal Masses - A Systematic Review. https://doi.org/10.6084/m9.figshare.31332268. ^33^ Data are available under the terms of the Creative Commons Attribution 4.0 International license (CC-BY 4.0).
