# Supplementary material for: Diagnostic Performance of Computed Tomography-Based Machine Learning Models in the Classification of Adnexal Masses - A Systematic Review
Source: F1000Res. 2026 Apr 2;15:464. [Version 1] doi: 10.12688/f1000research.178239.1 (PMC13596150; doi:10.12688/f1000research.178239.1)
Supplement: Peer review history

# Reviewer report: Reviewer response for version 1

**DOI:** 10.5256/f1000research.196606.r488044

**Published:** 2026-06-10

**Authors:** Priyanka Chandra Sekhar

**Affiliations:** 1 Imaging Technology, The Apollo University, Chittoor, Andhra Pradesh, India; 2 Sri Ramachandra Institute of Higher Education and Research (Deemed to be University), Chennai, Tamil Nadu, India

**Summary of the Article**

 This systematic review evaluates the role of computed tomography (CT)-based radiomics and machine learning (ML) models in the characterization of adnexal masses. The authors systematically reviewed the available literature and included 11 studies involving 4,439 patients. The review demonstrates that CT-based radiomics and AI models show promising diagnostic performance, with AUC values ranging from 0.72 to 0.99. The manuscript highlights the potential value of radiomics and deep learning approaches in differentiating benign and malignant adnexal masses and in supporting prognostic assessment.

**Overall Comments**

 The manuscript addresses an important and rapidly evolving area in gynecologic imaging. The review is well structured, follows PRISMA guidelines, and presents clinically relevant findings. The topic is timely and the conclusions are generally supported by the available evidence. The manuscript is suitable for indexing after minor revisions aimed at improving clarity, consistency, and methodological transparency.

 Minor Comments and Suggestions

1. Clarification of Study Selection

The manuscript states that 12 articles underwent full-text review, while 11 studies were ultimately included. It would be helpful to clearly mention the reason for exclusion of the remaining study within the PRISMA flow diagram or Results section to improve transparency.

 2.Language and Editorial Corrections

 A careful language review is recommended to correct minor grammatical and typographical issues. Examples include:

- “chine” should be revised to “China.”
- Minor inconsistencies in capitalization and punctuation should be corrected throughout the manuscript.
- Terminology Consistency

1. The terms “adnexal masses,” “ovarian tumors,” and “ovarian lesions” are used interchangeably throughout the manuscript. Consistent terminology would improve readability and avoid ambiguity.
2. Strengths of the Manuscript
  • Relevant and clinically important topic.
  • Well-organized systematic review following PRISMA guidelines.
  • PROSPERO registration enhances methodological rigor.
  • Comprehensive overview of recent CT radiomics and machine learning studies.
  • Appropriate use of QUADAS-2 for quality assessment.
  • Balanced discussion of current evidence and future applications.

Conclusion

- This manuscript provides a valuable overview of the current evidence regarding CT-based radiomics and machine learning models for adnexal mass characterization. The findings are clinically relevant and contribute to the growing body of literature in artificial intelligence-assisted imaging. The suggested revisions are minor and primarily intended to improve clarity, transparency, and overall presentation.

Are the rationale for, and objectives of, the Systematic Review clearly stated?

Yes

Is the statistical analysis and its interpretation appropriate?

Yes

If this is a Living Systematic Review, is the ‘living’ method appropriate and is the search schedule clearly defined and justified? (‘Living Systematic Review’ or a variation of this term should be included in the title.)

Not applicable

Are sufficient details of the methods and analysis provided to allow replication by others?

Yes

Are the conclusions drawn adequately supported by the results presented in the review?

Yes

Reviewer Expertise:

Radiology and Imaging Sciences

I confirm that I have read this submission and believe that I have an appropriate level of expertise to confirm that it is of an acceptable scientific standard.

# Reviewer report: Reviewer response for version 1

**DOI:** 10.5256/f1000research.196606.r473063

**Published:** 2026-05-14

**Authors:** Manna Debnath

**Affiliations:** 1 Charotar University of Science and Technology, Anand, Gujarat, India; 2 Radiography & Advance Imaging Technology, RSMAS, Royal Global University (Ringgold ID: 305831), Guwahati, Assam, India

**The author investigated the research entitled “Diagnostic Performance of Computed Tomography-Based Machine Learning Models in the Classification of Adnexal Masses - A Systematic Review”.**

**Comments**

1. Introduction and methods are well written.
2. In the Results section, under the selection process, it is stated that “12 were found to be eligible for full-text screening.” However, in the study characteristics section, it is mentioned that “this systematic review combines the results of 11 retrospective studies on CT radiomics.” Additionally, the PRISMA flowchart indicates that 12 studies were included in the review. This appears to be a minor discrepancy in the reported data. Kindly review and rectify this inconsistency.
3. In the Results section, under study characteristics (line 2), it is stated that “machine learning studies published between 2021 and 2025, with a total sample of 4,439 patients, were included.” However, when the sample sizes are summed from Table 1, the total appears to be 4,416. This indicates a discrepancy in the reported data. Please correct it.
4. The discussion and conclusions are well written.

Are the rationale for, and objectives of, the Systematic Review clearly stated?

Yes

Is the statistical analysis and its interpretation appropriate?

Yes

If this is a Living Systematic Review, is the ‘living’ method appropriate and is the search schedule clearly defined and justified? (‘Living Systematic Review’ or a variation of this term should be included in the title.)

Yes

Are sufficient details of the methods and analysis provided to allow replication by others?

Yes

Are the conclusions drawn adequately supported by the results presented in the review?

Yes

Reviewer Expertise:

Medical Imaging Technology, CT & MRI

I confirm that I have read this submission and believe that I have an appropriate level of expertise to confirm that it is of an acceptable scientific standard.
